# Ileosigmoid knotting: One of the largest single-center series

**DOI:** 10.12669/pjms.343.14893

**Published:** 2018

**Authors:** Sabri Selcuk Atamanalp

**Affiliations:** 1 Prof. Sabri Selcuk Atamanalp, MD. Department of General Surgery, Faculty of Medicine, Ataturk University, Erzurum, Turkey

**Keywords:** Ileum, Intestinal Obstruction, Knotting, Series, Sigmoid Colon

## Abstract

Ileosigmoid knotting (ISK) is the wrapping of the ileum or sigmoid colon around the base of the other structure, causing a double-loop intestinal obstruction. The disease generally presents as an intestinal obstruction with volvulus triad, including abdominal pain/tenderness, distention, and obstipation. Abdominal X-ray findings are not pathognomonic, and computerized tomography (CT) and magnetic resonance imaging (MRI) are more useful in the diagnosis. A patient with ISK generally requires an emergency laparotomy following resuscitation. Based on the viability of the ileum and sigmoid colon, different resectional or non-resectional surgical techniques may be used. In this report, one of the largest single-center ISK series in the world, an eighty-case series, is concisely presented.

## INTRODUCTION

ISK, the wrapping of the ileum or sigmoid colon around the base of the other structure, is an uncommon disease worldwide.^1^,^2^ As of 2018, less than 500 cases have been reported in the literature.^3^,^4^ Interestingly, the incidence of ISK is relatively high in eastern Anatolia,^5^-^7^ where our university clinic is located. Consequently, our ISK series is one of the largest single-center ISK series in the world according to the literature included major research databases, such as Web of Science^3^ and PubMed.^4^

In this paper, we present our clinical experience with 80 patients treated over a 51.5-year period between June 1966 and January 2018. The data were retrospectively analyzed until June 1986 and prospectively thereafter.

## CLINICAL EXPERIENCE

The incidence of ISK was 1.6 cases per year and 0.4 cases per 100,000 persons per year. The mean age was 47.5 years (range: 7-92 years). Of the patients, 58 (72.5%) were male. Abdominal pain (100.0%), obstipation (98.8%), distention (96.3%), and vomiting (77.5%) were the most common symptoms. Abdominal tenderness (100.0%), distention (96.3%), hypo/akinetic bowel sounds (62.5%), empty rectum (50.0%), guarding/rebound tenderness (47.5%), hyperkinetic bowel sounds (27.5%), and melanotic stool (15.0%) were the most common signs. Of the patients, 53.8% were in a shock state. Plain abdominal X-ray findings, including a dilated sigmoid colon with multiple intestinal air-fluid levels, were observed in only 7.0% of patients. When used, CT and MRI findings, including whirled and knotted sigmoid colon and terminal ileum mesenteries with a dilated sigmoid colon and multiple intestinal air-fluid levels, were observed in 100.0% (9/9 and 3/3, respectively) of patients. The preoperative accurate diagnosis rate was 13.8%. Misdiagnoses, including non-specific intestinal obstruction and non-obstructive acute abdomen, were noted in 68.8% and 17.5% of patients, respectively.

At laparotomy, bowels were viable in 19 patients (23.8%) and gangrenous in the remaining 61 (76.3%), including ileum gangrene in eight, sigmoid colon gangrene in seven, and double-segment gangrene in 46. In non-gangrenous patients, detorsion was used in 14 patients, sigmoid mesopexy was applied in two, sigmoid resection with primary anastomosis in two, and sigmoid resection with colostomy in one. In patients with ileal gangrene, after the resection, primary anastomosis was performed in seven patients, and ileostomy was performed in one. In patients with a gangrenous sigmoid colon, colostomy was applied in 6 patients, and primary anastomosis was applied in one following the resection. In double-segment gangrenous patients, after the resection, ileal primary anastomosis with colostomy was used in 38 patients, sigmoid primary anastomosis with ileostomy in three, and double segment stoma in three. In the last group, two patients died during laparotomy. The mortality was 18.8% with the highest mortality rate in the double-segment gangrenous group (28.3%). The morbidity was 20.0% with the highest morbidity rate in the same group (28.3%).

## DISCUSSION

Although ISK is rarely observed worldwide^1^,^2^, it is accepted as an endemic disease in Turkey, particularly in our region Eastern Anatolia.^5^-^7^ As presented in our series, high altitude, high-fiber diet habits, male gender and advanced age are known as probable causes of an anatomical predisposition to ISK, namely, an elongated sigmoid colon with a narrow mesentery in addition to a hypermobile terminal ileum.^1^,^2^,^5^,^8^

The classical volvulus triad, abdominal pain/tenderness, distention, and obstipation, is observed in 27.3-100% of patients.^1^,^2^,^5^,^7^-^14^ X-ray features, such as a dilated sigmoid colon with multiple intestinal air-fluid levels, most commonly indicate a sigmoid volvulus or a nonspecific intestinal obstruction.^1^,^2^,^5^,^8^,^9^ CT and MRI findings, including a knot in the ileum and sigmoid mesenteries with a dilated sigmoid colon and multiple intestinal air-fluid levels, is highly diagnostic in greater than 90% of cases.^1^,^2^,^5^,^9^,^11^

Given that endoscopic detorsion will likely be unsuccessful due to the double-loop knot and is hazardous given the possibility of missing ileal gangrene, endoscopic decompression is generally not attempted in ISK. After an early and effective resuscitation, an emergency laparotomy is needed. In non gangrenous cases, detorsion alone can be used with a mean rate of 1-5% for mortality and 5-15% for morbidity. In addition, a volvulus preventing procedure, such as sigmoid mesopexy or mesoplasty, can be added with a mean rate of 1-8% for mortality and 10-20% for morbidity, whereas sigmoid resection with anastomosis may be performed with a mean rate of 1-10% for mortality and 15-25% for morbidity to prevent sigmoid volvulus recurrence in some selected cases with good conditions.^1^,^2^,^6^-^16^ Unfortunately, gangrenous bowel develops in approximately 73.5-90.9% of ISK cases.^1^,^2^,^6^-^14^ In gangrenous cases, after the resection, primary anastomosis may be performed with a mean rate of 5-30% for mortality and 10-40% for morbidity in some patients with good conditions. In addition, resection with colostomy or ileostomy may be lifesaving in selected patients with a poor general condition or with a bowel problem, such as perforation, ischemia, edema, or difference in proximal and distal bowel diameters, for which the mean rates for mortality and morbidity are 20-60% and 30-80%, respectively.^1^,^2^,^6^-^16^ In our opinion, if a stoma is needed, the procedure should be applied only for one segment, which has one of the poor conditions abovementioned, rather than a double-segment stoma. If possible, we always prefer a colostomy instead of an ileostomy.

## CONCLUSION

The results of the present series, which are compatible with literature data, have an important role in data production for ISK. Although our ISK incidence observed a decrease in recent years, in our opinion, our series will contain the greatest number of ISK subjects for many years.
